# The Zebrafish as a Model Host for Invasive Fungal Infections

**DOI:** 10.3390/jof4040136

**Published:** 2018-12-13

**Authors:** Emily E. Rosowski, Benjamin P. Knox, Linda S. Archambault, Anna Huttenlocher, Nancy P. Keller, Robert T. Wheeler, J. Muse Davis

**Affiliations:** 1 Department of Medical Microbiology and Immunology, University of Wisconsin-Madison, Madison, WI 53716, USA; erosowski@wisc.edu (E.E.R.); bpknox@wisc.edu (B.P.K.); huttenlocher@wisc.edu (A.H.); npkeller@wisc.edu (N.P.K.); 2 Department of Molecular and Biomedical Sciences, University of Maine, Orono, ME 04469, USA; linda.archambault@maine.edu; 3 Department of Pediatrics, University of Wisconsin-Madison, Madison, WI 53792, USA; 4 Department of Bacteriology, University of Wisconsin-Madison, Madison, WI 53706, USA; 5 Graduate School of Biomedical Sciences and Engineering, University of Maine, Orono, ME 04469, USA; 6 Stead Family Department of Pediatrics, Carver College of Medicine, University of Iowa, Iowa City, IA 52242, USA

**Keywords:** zebrafish, *Candida*, *Aspergillus*, *Cryptococcus*, mucormycosis, *Talaromyces*, macrophage, neutrophil, innate immunity, host–pathogen interactions

## Abstract

The zebrafish has become a widely accepted model host for studies of infectious disease, including fungal infections. The species is genetically tractable, and the larvae are transparent and amenable to prolonged in vivo imaging and small molecule screening. The aim of this review is to provide a thorough introduction into the published studies of fungal infection in the zebrafish and the specific ways in which this model has benefited the field. In doing so, we hope to provide potential new zebrafish researchers with a snapshot of the current toolbox and prior results, while illustrating how the model has been used well and where the unfulfilled potential of this model can be found.

## 1. Introduction

Although we have been aware of human pathogenic fungi for over 100 years, they remain a worsening problem with the rising immunosuppressed population—a consequence primarily of HIV and iatrogenic immunosuppression [1]. Beyond the opportunistic infections, several notable fungal pathogens such as *Blastomyces* and *Cryptococcus gattii* prey upon apparently immunocompetent hosts equally well [2]. Yet, in the face of an expanding burden of fungal disease, our options for treatment have had abysmal growth in comparison to the expansion of antimicrobials directed toward bacterial or even viral pathogens [3,4,5]. Over the past decade or so, as antimicrobial resistance has expanded, more emphasis has been placed on treatments directed at targets specific to the host–pathogen interaction, whether this means products of the host or of the microbe. Such approaches in theory would allow these new drugs to have minimal impact on the survival of commensal organisms, and thus be less prone to driving universal resistance in the microbiome. In order to apply this approach to fungal infections we will need an intimate understanding of host–pathogen interactions down to the molecular level.

While traditional mammalian models such as mice and rats have the advantage of relatively close similarity to humans, they have limits in terms of complete identity with the human immune system and poor visual access to events of pathogenesis at the cellular level. The development of tissue culture methods which allow direct observation of human cells with pathogenic organisms has helped to fill the gap in many ways, but it is inherently challenging to use this system to study the interaction of multiple cell and tissue types. In addition, aside from experiments with freshly harvested primary cells, tissue culture remains somewhat artificial in the use of standardized cell line preparations (such as immortalized lines or induced differentiation of macrophages, dendritic cells, etc.), resulting in large numbers of cells which may or may not truly reflect the biology of naturally developing cells in vivo.

The zebrafish larva as a model host has been gaining acceptance as a way to fill the gaps between traditional mammalian models and cell culture. After this species became established as a genetically tractable model for vertebrate embryonic development, it was realized that its small size, fecundity, and compatibility with live microscopy gave it unique advantages as a model host for the study of host–pathogen interactions. A single mating can produce clutches of several hundred embryos, which survive on nutrients in the yolk for five to seven days or more [6]. By 36 h post fertilization (hpf), they have a functioning circulatory system which feeds a variety of distinct tissue types and supports an innate immune system including cells distinguishable as macrophages and neutrophils along with a fully functional complement system [7]. Larvae (as they are called after 72 hpf [8]) are transparent except for an array of pigment cells, the development of which can be genetically or chemically inhibited. Because developing larvae can easily be immobilized for microscopy from any angle, even multiple days in a row, infecting organisms can be followed throughout the body, allowing for an unprecedented view of dissemination and pathogenesis.

The larva, along with its adult counterpart, has now been used to model a variety of human infectious diseases from bacterial (mycobacteria, *Salmonella*, *Pseudomonas*, *Streptococcus*), to viral (spring viremia of carp, specific flaviviridae), to fungal to parasitic [9,10,11]. In this review we will focus specifically on the application of this model host to fungal infections, including the progress made to date and the specific areas in which this model will be uniquely useful in the future. Zebrafish models of some of the most prominent pathogenic fungi in humans have been developed, including *Candida*, *Aspergillus*, and *Cryptococcus* species, along with the broad group of filamentous fungi responsible for the clinical entity known as mucormycosis [1]. A study of *Talaromyces marneffei* (formerly *Penicillium marneffei*) has also recently been published [12]. Though these fungi are generally considered to be pathogens of the immunocompromised, this is not always true. Most characteristically however, they tend to be seen in specific host–pathogen contexts. For example, some forms of aspergillosis are particularly associated with chronic granulomatous disease [13]. Invasive candidiasis, aspergillosis and mucormycosis are classically seen in hosts with impaired innate immune function, especially neutropenic patients. Severe forms of mucocutaneous candidiasis and cryptococcal pneumonia and meningitis, on the other hand, have a proclivity for hosts lacking appropriate adaptive immunity, notably those with AIDS or with prolonged immune suppression as after solid organ transplant [1]. The reasons why these particular pathogens and syndromes appear in the context of specific immune deficits poses not only a puzzling and clinically relevant question but also a unique opportunity to learn more about the functions of the immune system itself. The lack of a functioning adaptive immune system in the larval zebrafish means that this model host is best used for studying the innate immune system in isolation, allowing for a new understanding of a surprisingly ‘adaptable’ innate immunity. To introduce each of the major fungal infections modeled in the zebrafish, we will start with findings related to phagocyte–pathogen interactions.

## 2. Phagocyte Function and Interactions with Fungal Pathogens

Larval zebrafish provide an ideal model in which to investigate phagocyte–fungal pathogen interactions due to the ease of live imaging. The small size of the zebrafish larva makes diffusion sufficient for gas exchange thus providing opportunities to view host cell–pathogen interactions in vivo over long time periods. Additionally, living larvae can be imaged for multiple days in a row to follow the entire progression of an infection, another unique advantage of the system. While fungal infection models in mice can require large fungal inocula in order to later locate and image phagocyte–pathogen interactions, in larval zebrafish infections of <10 fungal propagules can be located and visualized without harm to the host. To visualize the host side of these interactions, multiple established zebrafish lines exist which label neutrophils and macrophages or mark inflammatory gene expression [14,15,16,17,18,19,20].

A plethora of models of immune deficiency are also established in the zebrafish (Table 1). In particular, established genetic and experimental methods for specific depletion of macrophages or inactivation of neutrophils allow for the specific contributions of these cell types to be elucidated. Depletion of macrophages can be achieved through injection of a low dose of morpholino against Pu.1 [21], clodronate liposome injection [22] or mutation/knockdown of the transcription factor Irf8, which is required for early differentiation of macrophages from myeloid progenitors [23]. However, *irf8*^−/−^ larvae also possess more neutrophils than wild-type larvae, complicating conclusions drawn from experiments in this line [23]. The nitroreductase system is also used in zebrafish to ablate specifically either neutrophils or macrophages [24]. Two human genetic diseases associated with neutrophil function, WHIM (warts, hypogammaglobulinemia, infections, and myelokathexis), and LAD (leukocyte adhesion deficiency) type IV, have been modeled in the zebrafish [25,26]. As seen in human patients, neutrophils in the correlating lines of mutant zebrafish display an inability to migrate to sites of tissue damage. Both of these lines can be used to model infection in neutrophil-defective hosts.

### 2.1. Aspergillus

A larval zebrafish model of *Aspergillus fumigatus* infection was first published in 2014 [27]. Knox et al. injected ~50 spores of the *A. fumigatus* clinical isolate Af293 into the hindbrain ventricle of larvae, finding that infected wild-type larvae exhibited limited fungal growth and minimal death. Immune-deficient larvae lacking phagocytes were, on the other hand, extremely susceptible to infection due to pervasive invasive growth of the fungus. This initial model therefore recapitulated the human scenario—resistance of healthy individuals and susceptibility of immunosuppressed patients. Subsequent studies have recapitulated these results [31,35], while another found significant mortality in the wild-type larvae, a difference that is likely due to the use of a different clinical isolate [36] (see discussion of strain variation below). *Aspergillus* spores have also been injected via intramuscular and intravenous routes, producing similar results [12,31].

Other forms of immune compromise that can be modeled in the zebrafish (Table 1) are highly relevant to specific human patient scenarios. Calcineurin inhibitors such as FK506 or cyclosporine are commonly used to immunosuppress solid organ transplant patients, causing these patients to be at high risk for invasive aspergillosis [30,37], and Armstrong-James and colleagues modeled this immunosuppression in zebrafish infected with *A. fumigatus* by adding FK506 to the larval water [36,38]. The authors found that this treatment increases mortality after *A. fumigatus* infection, due to a decreased inflammatory response and increased fungal growth. Rosowski et al. also found that a different immunosuppressive drug, the corticosteroid dexamethasone, increases the susceptibility of larval zebrafish to *A. fumigatus* [31].

In addition to studying the impact of absent or altered phagocyte function, studies of *Aspergillus* infection in larval zebrafish have helped to elucidate the differential response of and requirement for neutrophils and macrophages. In a neutrophil-defective line, 50–90% of larvae die by seven days post injection (dpi) [27,31,35], while in larvae depleted of macrophages through clodronate liposome injection, ~90% succumb to infection [31]. However, in *irf8*^−/−^ larvae, in which the first appearance of macrophages is delayed [23], only ~20–40% mortality is observed [27,31]. A major difference between clodronate liposome-injected and *irf8*^−/−^ larvae is the number of neutrophils they possess—in *irf8*^−/−^ larvae, many myeloid progenitor cells become neutrophils instead of macrophages. Therefore, these data suggest that an increase in neutrophils can partially compensate for a lack of macrophages in an innate immune response to *A. fumigatus*.

The differential involvement of macrophages and neutrophils is directly related to the developmental stages of the fungus, which converts from conidia to filamentous hyphae over time. Knox et al. found that macrophages arrive first to the site of *A. fumigatus* infection in immune competent larvae, phagocytosing the majority of conidia within six hours [27]. Germination of *A. fumigatus* into hyphae was rarely seen in the immunocompetent animals, but by screening many infected larvae, the authors found that neutrophils only responded post-germination [27]. The initial recruitment of macrophages, followed by later recruitment of neutrophils, was confirmed by both Herbst et al. and Ellet et al. [12,30]. Macrophages that do not kill ingested spores primarily inhibit *A. fumigatus* germination, which in some cases can provide a temporary protective niche for *A. fumigatus* spores [31]. While the majority of phagocytosed *A. fumigatus* spores are killed by macrophages in vitro [39,40], Knox et al. reported that in wild-type larval zebrafish, *A. fumigatus* can persist for >7 days, with a significant decrease in the fungal burden only observed starting at 5 dpi [27]. Similarly, another group found that macrophages cannot always control *A. fumigatus* growth, and observed lateral transfer of spores to other macrophages [38]. After germination, hyphae can be targeted by both neutrophils and macrophages, and direct contact-mediated killing by neutrophils and macrophages has been observed in larvae [27,41]. In fact, *irf8*^−/−^ larvae infected with a fast-germinating strain of *A. fumigatus* exhibit less death and more fungal clearance compared to wild-type controls, underlining the ability of neutrophils to attack *A. fumigatus* post-germination [31].

**Figure 1:**
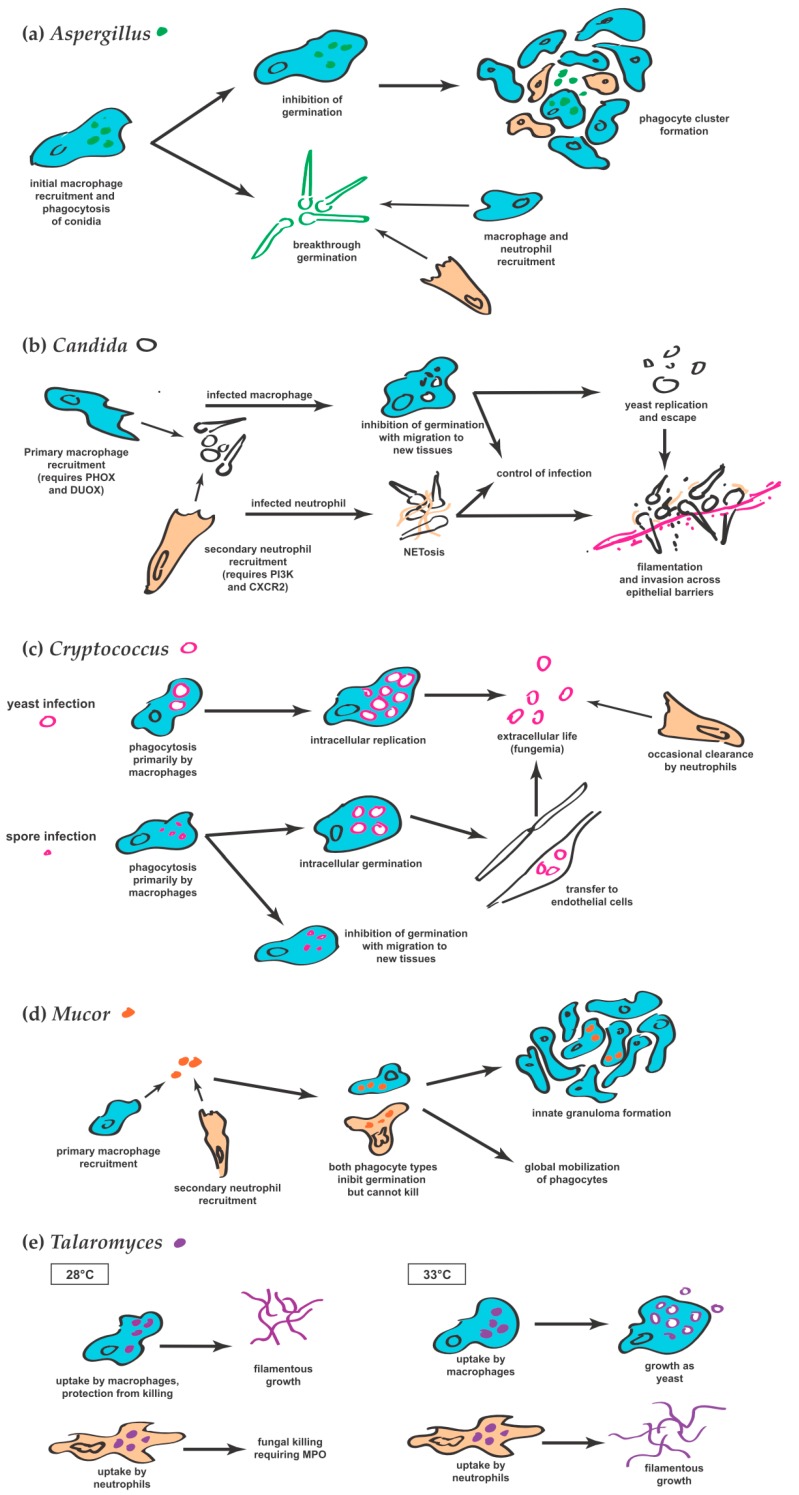
Diagrammatic depiction of the roles of macrophages and neutrophils during infection with (**a**) *Aspergillus*; (**b**) *Candida*; (**c**) *Cryptococcus*; (**d**) *Mucor*; and (**e**) *Talaromyces*. Macrophages are rendered in blue and neutrophils in orange. Spore and conidia forms of fungi are depicted with solid shapes while yeast forms are depicted as open shapes. Note the often-repeated themes of earlier macrophage than neutrophil recruitment, different fungal fates in macrophages versus neutrophils, inhibition of germination by one or both phagocytes, and fungal escape from the intracellular environment.

Rosowski et al. have recently extended these observations of phagocyte behavior by looking even later in infection, and reporting the formation of phagocyte clusters around the injected fungus starting 1–2 dpi [31]. These clusters persist for multiple days, but the number of neutrophils and macrophages within them are dynamic [31]. A graphical summary of the roles of neutrophils and macrophages in *Aspergillus* infection is shown in Figure 1a.

### 2.2. Candida

Over the past 10 years, work from several laboratories has established mucosal, disseminated, and localized infection models for *C. albicans* in zebrafish larvae, taking advantage of the transparency of the zebrafish larva to monitor single-cell dynamics over the long-term to understand determinants of infection progression, pathogenesis and immune response [28,29,42,43,44,45,46,47,48]. One conundrum in the field has been that macrophages are important in immunity to *C. albicans* in vivo, but when isolated macrophages ingest yeast, the fungi germinate and kill the macrophages rapidly and *efficiently* in vitro [49,50,51,52]. However, during *C. albicans* infection in the zebrafish it is clear that macrophages can prevent fungal germination and thereby provide a crucial brake on the infection. In fact, the efficiency of fungal ingestion within the first four hours of infection is crucial for overall survival [28]. The engulfed yeast were found to survive, divide, then exit macrophages far from the infection site [28] (Scherer and Wheeler, unpublished data), suggesting that *C. albicans* has a mixed intra-/extra-cellular lifestyle during infection that includes Trojan horse-mediated fungal dissemination.

Early phagocyte containment could be enhanced by opsonizing antibodies and is a crucial determinant of overall survival in the hindbrain ventricle infection model [28,53]. Experiments also found that macrophage recruitment to filamentation-competent *C. albicans* requires both phagocyte oxidase (Phox) and dual-specific oxidase (Duox), revealing a new role for NADPH oxidases in phagocyte recruitment [28]. In addition to phagocyte recruitment, zebrafish also allow for the assessment of phagocyte activation and recent work in both the swimbladder and yolk models of infection demonstrate that the major cell type expressing TNF-α at the site of infection is the macrophage, whose presence there drives large increases in TNF-α expression host-wide (A.K.S., L.S.A., and R.T.W., unpublished). Taken together, these studies in the larval zebrafish have expanded our appreciation for the versatility of *C. albicans*, identified a new role for NADPH oxidases in the immune response and highlighted the importance of rapid macrophage responses in limiting lethal infection.

Neutrophils engulf *C. albicans* yeast and attack filaments in both the hindbrain ventricle and swimbladder [28,29]. Neutrophilic attack drives production of extracellular traps in the swimbladder and limits hyphal penetration of the epithelial barrier, providing a crucial element to mucosal immunity [29]. This neutrophil response requires both PI3K and CXCR2, as in mammalian systems [29]. Extracellular neutrophil histones suggestive of neutrophil extracellular traps (NETs) were also observed after hindbrain infection with *C. albicans* [54]. See Figure 1b for a summary of macrophage and neutrophil roles in *Candida* infection.

Several reporter strains of *C. albicans* have also been used in larval zebrafish to probe the micro-environmental conditions of the fungi during infection. This has included sensors for oxidative stress [28], arginine starvation [55], and hyphal growth [53], revealing microclimates within the host to which the fungi are exposed. The ability to visualize infection has deepened our appreciation for mechanisms discovered in simplified cell culture interactions and has also sparked new hypotheses about fungal virulence and immunity that remain to be tested in mammalian systems.

### 2.3. Cryptococcus

In 2015 and 2016, three different groups published independent studies establishing the zebrafish larva as a model for cryptococcal infection [56,57,58]. These studies are essentially non-contradictory, although they each approached different aspects of infection. All three groups found a similar progression of pathogenesis after intravenous yeast infection. Macrophage interactions with cryptococcal yeast cells have long been considered a central theme in cryptococcal infection. Studies in the zebrafish are consistent with this theme, with macrophages being the host cell most commonly associated with the pathogen. However, in accordance with studies in other animal models, all three studies thus far have concluded that escape from macrophages is very common. The first of these studies [58] used the yeast form of the highly virulent H99 strain (see below for a discussion of strain differences) and several closely related mutants to demonstrate both how well the zebrafish model recapitulated mutant phenotypes seen in mammalian models, as well as to provide in vivo observations of yeast–phagocyte interactions. Using the PU.1 morpholino to inhibit macrophage development (Table 1), they demonstrated the central importance of macrophages in control of cryptococcal growth, and provided in vivo evidence of cryptococcal reproduction inside macrophages.

The second publication of cryptococcosis in zebrafish [56] used an entirely different approach, taking advantage of the zebrafish larva’s capacity for high-throughput imaging and analysis. Using infected larvae mounted in a 96-well format for repeat imaging at various stages of infection they produced a robust dataset of dose response and yeast–phagocyte interactions. They distinguished three distinct groups of infection doses based upon the extent of yeast replication by 72 hpi, as well as mortality after that time-point. As in the first paper, they demonstrated the importance of macrophages in control of fungal growth, confirming the findings of the first group using chlodronate liposomes to deplete macrophages. With the addition of daily quantification of yeast cells, the authors demonstrated the impact of delayed macrophage depletion, which was equally severe. The most elegant analysis of this dataset took advantage of a zebrafish line with fluorescently labeled macrophages to analyze the complex yeast–phagocyte interactions during the first 24 h of infection. In short, they were able to demonstrate that: (1) yeast switch from primarily extracellular to primarily intracellular life between 2 and 24 hpi, (2) this relative shift is due mostly to intracellular replication, as opposed to new phagocytosis events, (3) the increased morbidity associated with higher inocula was not due to exhaustion of macrophages, and (4) the yeast cells phagocytosed early in infection had thinner capsules than those which evaded phagocytosis. Lastly, by manipulating capsule size prior to inoculation they showed that the initial difference in capsules between these two groups was due at least in part to a qualitative factor, and not simply the volume of capsule present. This group was the first to demonstrate vomocytosis, the non-lytic expulsion of Cryptococci from macrophages, in vivo. Using intensive time-lapse imaging, they found excellent visual examples of the process and were able to quantify it in terms of the whole infection. It was found that 5–15% of observed macrophages underwent vomocytosis during a 12-h period. This work gave rise to a follow-up publication from the same institution, demonstrating that stimulating the process of vomocytosis reduced overall dissemination of infection. This is a fine example of how the visual access of the zebrafish can improve our understanding of cellular processes in pathogenesis and thereby open up new avenues for intervention.

The third of the articles from 2016 explored the distinctions between infections by the spore and yeast forms of *Cryptococcus* [57]. The authors chose a strain pair quite distinct from the H99 strain used by both other groups because of the ease with which they produce spores. Neither spore nor yeast form of this strain proved as virulent as H99 in terms of host survival. Still, both cell types survived and replicated intracellularly and often underwent considerable replication without killing the host within a seven-day observation period. With daily observations of infected larvae, they showed that both forms gradually progressed to produce a sustained, very low-level fungemia. Establishment and maintenance of this fungemia involves a continuous cycle of phagocytosis, intracellular survival, and eventual escape, with or without the death of the enclosing phagocyte—recapitulating the findings of vomocytosis first shown by Bojarczuk et al. [56]. Analysis of intracellular cryptococcal cells showed that most were contained within macrophages. Interestingly, inoculated spores (but not yeast) were shown to take up residence inside peripheral endothelial cells, raising the possibility of these cells providing a niche for long term survival. Similar to the findings of Tenor et al. [58], they also demonstrated that intravascular yeast and germinated spores lodged in the brain vasculature. Finally, using a pair of host mutants with decreased function or production of either neutrophils or macrophages, they demonstrated that neutrophils are very capable of killing cryptococcal cells in vivo and that their function is actually critical during the later, fungemic stages of infection. Figure 1c depicts the interactions between cryptococcal yeast and spores with macrophages and neutrophils.

### 2.4. Agents of Mucormycosis

Mucormycosis is a clinical term for the syndrome associated with a range of fungi, primarily consisting of *Rhizopus, Mucor, Lichtheimia* (formerly *Absidia*), *Cunninghamella, Rhizomucor, Apophysomyces*, and *Saksenaea* [59]. A zebrafish model of mucormycosis using strains of *Mucor circinelloides* has been established using both larval and adult zebrafish [32,60,61]. In zebrafish larvae, *M. circinelloides* can be more or less pathogenic depending on the site of infection. Voelz et al. showed that survival after hindbrain ventricle infection was around 60% with an inoculum of ~100 cfu, while the same inoculum in the swim bladder is not lethal [32]. The administration of dexamethasone increases mortality in both sites, but swim bladder infections remain less severe [32,60]. Interestingly, inoculated spores are not cleared and remain viable even in larvae which do not succumb to infection. One major finding in the zebrafish which contradicts other animal models and in vitro studies of mucormycosis is that sporangiospores elicit robust recruitment of phagocytes in zebrafish [32,60]. Ingelsfield et al. generated a mathematical model of the infection based on data from a large number of larvae, to conclude that of all factors the maximum number of recruited macrophages to the site of infection was most vital to survival.

Similar to the case in *Aspergillus*, macrophages arrive first, followed by neutrophils after a lag of about four hours (Figure 1d) [60]. While they are unable to clear viable spores, these cells can often prevent germination and thus ward off active infection. How macrophages and neutrophils inhibit germination remains unknown, but this inhibition is reversed by the administration of dexamethasone and is impaired in phagocytes from diabetic mice [62]. The formation of dense clusters of macrophages which the authors term ‘innate granulomas’ appears to be a key element of defense against this form of infection [60]. In other work using larval zebrafish, these types of early granulomas were found to be a site for replication and dissemination of the bacterial pathogen *Mycobacterium marinum* [63,64], suggesting a similar role for this structure in mucormycosis.

More recently, López-Muñoz et al. published their model of intraperitoneal mucormycosis in the adult zebrafish [61]. This study included broad analysis of gene expression changes during infection and new findings regarding the impact of overwhelming mucormycosis on hematopoiesis. In line with findings in zebrafish larvae, germination is not very widespread after inoculation with the same strain. However, a more virulent strain which produces larger sporangiospores germinated abundantly and rapidly led to host death. This also parallels findings in larvae using a different hypervirulent strain.

*M. circinelloides* infection affects global phagocyte populations, as demonstrated in both larval and adult zebrafish models of infection. Inglesfield et al. elegantly demonstrated that total body macrophage and neutrophil numbers increase during infection, beyond the developmentally normal increase over time [60]. In the adult zebrafish model, infection with the large, highly virulent spores of the R7B strain resulted in a marked decline in the number of myeloid cells present in the hematopoietic regions of the kidney [61]. This was interpreted as a large mobilization of these cells into the periphery. Finally, large numbers of peripheral macrophages were shown to have an apoptotic appearance, along with expression of caspase 3, representing the first evidence of induction of apoptosis as an aspect of virulence in mucormycosis.

### 2.5. Talaromyces marneffei (Formerly Penicillium marneffei)

The ability to raise larval zebrafish at different temperatures recently allowed Ellett et al. to investigate the effect of temperature on phagocyte responses to and the in vivo development of the thermally dimorphic fungus *Talaromyces marneffei* [12]. Injection of *T. marneffei* conidia at 28 °C resulted in phagocytosis by leukocytes (almost exclusively by macrophages), followed by filamentous growth of the fungus (Figure 1e). However, at 33 °C, a temperature at which the yeast morphological switch occurs in vitro, conidia taken up by macrophages do indeed primarily grow as yeast while those taken up by neutrophils instead grow in a filamentous form. This observation demonstrates that the specific intracellular environment of phagocytes can override thermal cues for fungal development. These phagocytes also have opposing effects on *T. marneffei* growth at 28 °C; neutrophils target the fungus through myeloperoxidase-dependent mechanisms, while macrophages can protect the fungus from killing. Ellet et al. also found that infection induces an increase in neutrophil and macrophage numbers, dependent on G-CSFR signaling.

## 3. Assessment of Fungal Virulence Determinants

While the state of host immunity is clearly a critical factor in fungal disease development [65], it is increasingly recognized that the fungal isolate itself plays an important role in pathogenesis. Thus, it is critical to understand the specific fungal genes and pathways responsible for pathogenesis through tolerance, adaptation, and growth within the harsh host environment, with the aim of uncovering potential drug targets. Therefore, the study of fungal mutants offers a valuable approach to dissecting complex host–pathogen interactions in vivo, including identifying specific fungal virulence factor–host factor interactions and their effect on host survival and fungal clearance. To date, all fungal mutants analyzed in the larval zebrafish model have recapitulated virulence attenuation observed in other model systems, offering the advantage of lower cost, high-throughput screens for identifying potentially targetable virulence factors. In some instances, unique features of the model have allowed additional insight into classic fungal mutants. A summary of fungal mutants examined in zebrafish infections is shown in Table 2.

### 3.1. Aspergillus fumigatus Mutants

As a saprobe, many virulence determinants of *A. fumigatus* likely evolved outside of a vertebrate host in the soil environment [13]. Therefore, many of the canonical virulence factors of *A. fumigatus* are related to core metabolism and nutrient acquisition. Two of these genes, *sidA* and *pyrG*, required for production of an iron siderophore and de novo UMP biosynthesis, respectively, have been confirmed to be virulence factors in fish, recapitulating mouse studies [27,31,66,85,86].

A feature unique to *A. fumigatus* compared to the other fungal pathogens addressed in this review is the production of secondary metabolites (SMs). With broad-range bioactivities, some of these small molecules have been shown to play roles in host–pathogen interactions [87,88]. LaeA is an *A. fumigatus* master transcription factor which regulates global production of SMs and impacts virulence in mice [67,89]. Larval zebrafish with specific cell deficiencies provided the opportunity to test the effect of LaeA-regulated SMs on neutrophils versus macrophages, and Knox et al. reported that a ∆*laeA* strain of *A. fumigatus* only has attenuated virulence in a macrophage-deficient host, suggesting that these SMs primarily target neutrophil behavior and function [27]. Further work has focused on identifying LaeA-regulated SMs which affect immune cell functions and virulence of the fungus in a vertebrate host [90]. In fact, a screen in larval zebrafish for fungal metabolites that inhibit neutrophil chemotaxis identified the LaeA regulated spore metabolite endocrocin [87].

The effect of two additional LaeA-regulated genes that were identified in a microarray analysis [91] has also been tested in fish in the context of fungal infection. MetR is a bZIP transcription factor required for sulfur and methionine metabolism in fungi and *metR* gene expression was significantly downregulated in a ∆*laeA* strain [69,91]. However, overexpression of metR in a ∆*laeA* background failed to rescue the ∆*laeA* virulence defect in the zebrafish macrophage-deficient model [69]. Another gene downregulated in the ∆*laeA* mutant was *aceA*, encoding a copper-responsive transcription factor, and deletion of *aceA* in the fungus resulted in better clearance by the host [68,91]. This result led the authors to investigate further the role of copper in host killing of *Aspergillus*. Copper can be utilized as an antimicrobial mechanism by host cells against fungal pathogens [92] and the combination of copper and Phox-generated hydrogen peroxide can create damaging hydroxyl radicals through Fenton chemistry [68]. In fact, this increased clearance of a ∆*aceA* strain was dependent on host Phox activity. The amount of copper in host cell phagosomes is partially controlled by the ATP7A transporter [93]. In humans and mice *ATP7A* mutation causes Menkes disease and mouse models of this disease are very sick, precluding infection studies [94]. However, this gene can be transiently knocked down with morpholino in larval zebrafish, and Wiemann et al. showed that ATP7A also contributes to clearance of *Aspergillus*, providing another example of the utility of the larval zebrafish in dissecting host pathways that contribute to fungal killing.

### 3.2. C. albicans Mutants

*C. albicans* has several virulence attributes, but perhaps the most interesting of these is its ability to switch between yeast and filamentous growth phases during infection. Zebrafish models of candidiasis have been used largely to test the impact of this virulence factor, although some work has also gone into testing the differential pathogenesis of white and opaque switching morphologies. It is clear that filamentous growth is crucial for tissue invasion, damage and rapid lethality of *C. albicans* in larval, adult, and egg models of infection [29,42,44,45,48]. Yeast growth, on the other hand, is specialized for dissemination from the tissue into the bloodstream and throughout the host [48]. *C. albicans* cells that are not heterozygous at the mating-type loci are competent to switch between white and opaque forms [95]. White cells are in general more virulent, although opaque cells have some enhancement in virulence at lower temperatures, something that has only been testable in the larval zebrafish because of its flexibility in body temperature [82]. Interestingly, opaque cells are poorly phagocytosed by mammalian and zebrafish phagocytes both in vitro and in vivo, which would potentially allow them to avoid phagocytic containment and permit them to grow as potentially pathogenic filaments [82].

### 3.3. Cryptococcus neoformans Mutants

Analysis of several cryptococcal mutants was undertaken by Tenor et al. [58]. Each of the mutant yeasts tested in this study produced results similar to those described in mammals. The acapsular mutant *cap64Δ* was attenuated in virulence although it was still capable of florid growth in the zebrafish. Mutants lacking function of *plb1* (phospholipase B) or *tps1* (an element of the trehalose pathway) both were deficient in replication and causing host mortality, demonstrating that these factors of pathogenesis are operant at both mammalian and poikilothermic temperatures. Finally, the *ure1Δ* mutant (with loss of urease function) had little trouble replicating in the larvae but, like *cap64Δ*, was deficient in host killing. A particularly striking finding in this paper was the capacity for cryptococcal cells to disseminate to the brain through the vasculature by 4 dpi. The authors showed that *fnx1Δ*, a mutant strain deficient in crossing the blood-brain barrier in mice, was able to access the vasculature but not to invade neural tissues in the zebrafish, instead replicating vigorously inside the brain ventricles.

## 4. Assessing the Influence of Fungal Strain Heterogeneity upon Virulence

Natural strain variation observed within a species can impact the outcome of host–pathogen interactions. Studies of diverse, naturally occurring isolates have the benefit of providing a snapshot of phenotypic variability that could be taken into consideration when assessing at-risk environments and risk factors for disease susceptibility. Significant strain variation in drug susceptibility [96,97], stress tolerance [98], growth rate [99], and other clinically relevant phenotypes [100] have all been observed and even shown to affect host immune responses and survival [101,102]. The zebrafish model, with its unique imaging capability and suitability for high-throughput experimental approaches, is ideally positioned to assess the effect of strain variation on infection outcome. A summary of fungal strains used in the zebrafish is shown in Table 3.

### 4.1. Aspergillus fumigatus Strain Variation

Clinical and environmental isolates of *A. fumigatus* vary widely, with 147,792 total SNP positions found among 95 sequenced strains [35]. Two strains have emerged as common laboratory strains, Af293 and CEA10, and these strains differ by ~50,000 SNPs [35] and have significant phenotypic differences, including tolerance of hypoxia and response to light [98,107,108]. How strain differences correlate with differences in immune response and virulence remains only partially answered by mouse models [98,109]. Knox et al. first demonstrated the applicability of the zebrafish model to assess differences in strain virulence through infection of larval zebrafish with Af293, CEA10, or two *A. fumigatus* isolates found on the International Space Station. In a neutrophil-defective host, they found that these “space isolates” caused more mortality than either Af293 or CEA10, while CEA10 was also significantly more virulent than Af293 [35].

Rosowski et al. recently expanded these studies using the larval zebrafish model to compare the immune response to Af293 and CEA10 and the virulence of these strains in different host immunosuppressed backgrounds [31]. In addition to recruiting significantly more macrophages and neutrophils and inducing more immune pathway activation, CEA10 was more virulent than Af293 in a neutrophil-defective host, consistent with previous observations. However, CEA10 did not have increased virulence in macrophage-deficient hosts. In fact, in both wild-type larvae and *irf8*^−/−^ larvae, CEA10 fungal burden was cleared significantly faster than Af293 fungal burden. Rosowski et al. found that this increased clearance was directly driven by increased fungal germination of CEA10. This study in larval zebrafish therefore further elucidated the double-edged sword of *A. fumigatus* germination—while germination is required for fungal growth and propagation, it simultaneously activates the immune system and makes the fungus more susceptible to immune cell-mediated killing [110]—and the importance of understanding strain variation in the development of future antifungal therapies.

### 4.2. Candida Species Variation

Recently, a larval zebrafish model was used to elucidate differences in pathogenesis between *C. albicans* and the emerging pathogen *Candida auris*. *C. auris* is a major global public health threat, causing outbreaks of candidiasis with mortality rates close to 60%, but the pathogenesis of this infection is largely unknown [111]. In particular, *C. auris* is atypical among the pathogenic *Candida* species in that neutropenia is not a significant risk factor for disease. Johnson et al. therefore investigated the differential neutrophil response to these two species. In vitro studies with human neutrophils showed that *C. auris* is not readily taken up by neutrophils, nor did it induce the production of neutrophil extracellular traps (NETs) [54]. Parallel studies in the zebrafish confirmed that the neutrophil recruitment is much less robust in response to *C. auris* than *C. albicans* in vivo, and found evidence suggestive of NETosis only in response to *C. albicans*, not *C. auris* [54].

Interestingly, *C. parapsilosis* tends to provoke little to no immune response to epithelial cells and can even block responses to *C. albicans*. Consistent results were found in both an in vitro epithelial-*Candida* challenge model and in the zebrafish swimbladder mucosal disease model, suggesting that secreted products from *C. parapsilosis* block epithelial immune responses to *C. albicans* [112]. When host responses to *C. albicans* and *C. parapsilosis* were compared in the swimbladder mucosal infection model, *C. albicans* activated the NF-κB pathway, evoked pro-inflammatory cytokines, and caused the recruitment of phagocytic immune cells while *C. parapsilosis* remained in yeast morphology and caused recruitment of phagocytes without inducing inflammation. In spite of the differences in cytokine milieu, high resolution mapping of phagocyte-*Candida* interactions revealed that neutrophils and macrophages attack both *Candida* species (L.S.A. and R.T.W., unpublished).

### 4.3. Cryptococcus Strain and Species Variation

The pathogenic cryptococci have been subdivided in several ways in the past. The three groups of pathogenic cryptococci have recently been known as *C. neoformans* var. *grubii* and var. *neoformans* (previously named serotypes A and D respectively) and *C. gattii* [113]. *C. gattii* has been considered distinct from the other two, based upon epidemiological differences. To our knowledge, no experiments with *C. gattii* in the zebrafish have been published. Recently, it has been proposed [114] that *grubii* and *neoformans* varieties be divided into distinct species—*C. neoformans* and *C. deneoformans*, respectively, but it is unclear how universally this nomenclature will be accepted. For clarity we will call them *var. grubii* (*C. neoformans*) and *var. neoformans* (*C. deneoformans*) in this review. Clinico-pathological distinctions between these two groups have long been quite muddled. Of the two, *var. grubii* (*C. neoformans*) accounts for far more clinical isolates [104]. Within and between these groups however there is considerable strain to strain variation. This has complicated systematic study of cryptococcal pathogenesis due to mixed results depending upon which species or strain is being used. Of the three publications to date featuring cryptococcal infection of zebrafish, two [56,58] have used the yeast form of an especially virulent strain of *var. grubii* (*C. neoformans*) named H99, while the third used spores and yeast of a *var. neoformans* (*C. deneoformans*) mating pair (B3501 and 2), [57]. The H99 strain is easily more lethal to zebrafish larvae, but this is not entirely explained by its more rapid replication in vivo. With sufficient inocula, both H99 and B3501/2 can produce overwhelming fungemia in the zebrafish, but such overgrowth of B3501/2 can be tolerated for days before the larvae succumb (J.M.D., unpublished observations and [57]). Similar overgrowth of H99 yeast produces widespread vascular congestion which appears to be the cause of death. A second *var. neoformans* (*C. deneoformans*) strain pair (JEC21/22) does not grow as rapidly as H99 but causes a similar vascular congestion and lethality when it does overgrow (J.M.D., unpublished). Thus, the zebrafish model is capable of resolving fine differences in pathogenicity between strains and species in this complex and varied group.

### 4.4. Agents of Mucormycosis: M. circinelloides Strain Variation

As with *A. fumigatus*, there is documented evidence that strain heterogeneity can impact virulence in experimental infection models. In both murine [115] and larval zebrafish [32] models of infection, *M. circinelloides* strain CBS277.49 was more lethal in comparison to NRRL3631. Interestingly, CBS277.49 produces larger spores with spore size correlating with virulence among other isolates as well [115], making this readily observable phenotype a strong predictor of virulence. Spore size in *M. circinelloides* also correlates with mating type, (+) or (-), providing evidence that in this species an isolate’s mating type can be a predictor of virulence potential, which stands in contrast to speculation that mating type, MAT1-1 or MAT2-1 may play a role in predicting virulence in *A. fumigatus* [35,116]. The correlation with large spores and increased virulence was further supported using adult zebrafish by López-Muñoz et al. [61], who used RB7, another large spore strain, in comparison again with NRRL3631. As with CBS277.49 in the larval studies, RB7 proved markedly more virulent in intraperitoneal infections of adults.

## 5. Unique Aspects of the Zebrafish Toolbox: Infection Localizations

**Figure 2:**
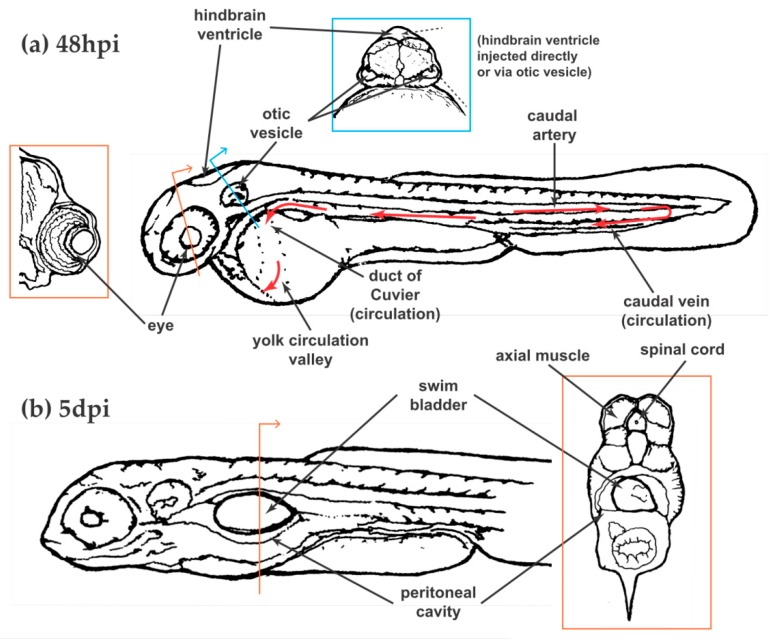
Infection localizations available in the zebrafish larva at (**a**) 48 hpi and (**b**) 5 dpi. Insets are included for cross-sectional information about eye, swim bladder, and intraperitoneal injection sites. Red arrows indicate direction of blood flow. The caudal artery is indicated for clarity but is not a preferred (intentional) site for injection into the circulation. Overall larva images redrawn from [8]. Cross-sectional images redrawn from [117].

Like any vertebrate model organism with defined structures and organs, the larval zebrafish model offers multiple routes and localizations for infection with the added benefit of being able to monitor whole-body consequences following infection at different localizations (i.e., dissemination). Of course, zebrafish lack certain organs that are critical in host-fungal interactions, such as lungs and the specialized pulmonary epithelium, making model selection important to support the question at hand. Figure 2 illustrates the primary infection localizations used to date in modeling fungal disease.

### 5.1. Circulation

Bloodstream infections have been the most commonly used in zebrafish larvae, in part because of the ease of injection, and in part because of the clear parallel this route offers to other animal models. Intravenous inoculations are performed via either the tail vein or the duct of Couvier (Figure 2a). While the tail vein is directly adjacent to hematopoietic tissue which can lead to injected pathogens lodging amongst the developing blood cells, the duct of Couvier drains into the yolk circulation valley (YCV), right over the surface of the yolk, providing excellent visual access. With appropriate positioning of the larva, this site is viewable using even very short working distance objectives, allowing unparalleled fluorescence and widefield microscopy of live phagocytes and pathogens in the unaltered circulation of a living vertebrate [118,119]. Of note, the best visual access to the YCV is between 24 and 32 hpf, but because the phagocytes at that time are not all fully differentiated into either macrophages or neutrophils, the 48 hpi time point is much more commonly used [120].

For the study of invasive fungal infections, inoculations into the circulation can be both a blessing and a curse. Except for *Candida*, none of the major fungal pathogens we focus on here typically begin pathogenesis in the human bloodstream. *Aspergillus*, *Cryptococcus*, and the agents of mucormycosis are thought to initiate infection in the lower airways, or occasionally through breaks in the skin. Subsequent stages of dissemination in the latter two certainly involve the bloodstream, but study of the events leading up to this is not ideally undertaken using this approach.

### 5.2. Yolk

Injection into the embryonic zebrafish yolk sac can also lead to systemic infection and injection into this site is similarly straightforward. The yolk sac provides nutrients to the developing larva and is surrounded by the yolk syncytial layer (YSL) which separates the yolk from the fish body proper [121]. Early work showed that phagocytes were recruited to this site in response to sterile injury and to *C. albicans* infection [44,122]. These phagocytes likely arrive through a network of blood vessels in close proximity to the yolk which may additionally give pathogens potential routes of dissemination [123]. As discussed above, the yolk can be an excellent location for detailed imaging of phagocyte-fungal interactions.

In a recent study, this injection site was used in zebrafish with macrophages expressing a photo-switchable reporter to show macrophages suffered a different fate after interacting with *C. albicans*. Once they had interacted with the pathogen, macrophages tended to remain in the infection area and were more likely to die than “bystander” macrophages [124]. Currently, the yolk infection site also is being used to dissect the complex web of host and pathogen factors contributing to the dissemination of *C. albicans* [48] (Scherer et al., manuscript in preparation).

### 5.3. Hindbrain Ventricle

The hindbrain ventricle (HBV) is a discreet fluid-filled cavity well-developed for microinjection at 36–48 hpf. Injection into the HBV is relatively easy, allowing for high-throughput survival analysis. Furthermore, this location is ideal for visualizing and quantifying phagocyte recruitment as the HBV is typically devoid of macrophages and neutrophils but these cells can easily migrate into the site This ability to image fungal–phagocyte interactions is a major strength of the HBV injection site as the HBV is separated from the environment only by a thin epithelial layer [28,41]. However, as the larva develops, the volume of this space decreases and injection and imaging becomes more difficult. Furthermore, while larvae do develop a functional blood–brain barrier [125], there is no clear clinically relevant parallel for the HBV transiently fluid-filled space. However, the capabilities of this model have revealed novel biology including differential phagocyte recruitment and behaviors towards fungi across morphological development [28,41].

### 5.4. Swimbladder

The zebrafish swimbladder is a mucosal, epithelial-lined, air-filled organ that functions to maintain and adjust buoyancy. The swimbladder displays homology with the mammalian lung in terms of anatomy, development, and gene expression [126,127,128,129], providing a clinically relevant model for fungal infections of the lungs such as *Aspergillus* and *Cryptococcus*. While the larval zebrafish swimbladder lacks the complexity of mature mammalian mucosal epithelial surfaces, such as the tongue or intestine, ease of injection and imaging make it a suitable general model and a good bridge between cell culture and mammalian models.

The zebrafish inflates the swimbladder with air at around 4.5 days post-fertilization (dpf), and injection of microorganisms into the swimbladder lumen is a relatively simple endeavor. Interactions between pathogens and host cells can be easily monitored at this site, as it is patrolled by phagocytes of the innate immune system which are recruited in greater numbers in response to infection, LPS and sterile injury [47,130]. The air bubble creates a clear optical background which enhances imaging at the inner surface of the swimbladder. However, the air bubble also makes the swimbladder the thickest part of the larva, so imaging is usually limited to one half the width of the swimbladder, and the relatively later age at which the swim bladder develops limits experiments to later larval stages. The entire organ can be dissected for clearer imaging of interactions between host epithelial cells, phagocytes, and fungal yeast or hyphae [46,47].

One example of an advance made possible by the swim bladder model, and the ability to longitudinally track infection progression and outcome in individual zebrafish larvae, is a study of tri-kingdom interactions between *C. albicans*, *P. aeruginosa* and the zebrafish host [105]. These pathogens are often co-isolated in human lung infections, particularly in cystic fibrosis patients. Bergeron et al. found a synergistic interaction between the two pathogens that led to elevated inflammation, as indicated by higher IL-6 expression and swimbladder edema, and increased *C. albicans* pathogenesis that manifested in greater fungal burden and more frequent epithelial invasion by fungal hyphae.

### 5.5. Eye

Zebrafish eye development is similar to that in humans and larval zebrafish are used in studying both corneal and retinal development [131], raising the intriguing possibility that the larval eye could hold potential as a model for fungal infections of the eye. These infections are a major cause of blindness compounded by difficult diagnosis and treatment through topical application of antifungals. Incidences of fungal keratitis are particularly prevalent in developing countries and military personnel where eye trauma is common and can occur in otherwise immunocompetent individuals. The most common etiological agents of fungal keratitis are the filamentous fungi, particularly *Aspergillus* and *Fusarium* species. While current murine models have provided a valuable platform for modeling *Aspergillus* keratitis [132], this model is not very amenable to tracking fungal–phagocyte interactions in vivo. While no published reports for a larval zebrafish fungal keratitis model currently exist, our labs have explored the developing eye for feasibility of microinjection with *A. fumigatus* spores. Injection into the space between the lens and neural retina resulted in macrophage phagocytosis in wild-type larvae and extensive hyphal invasion in larvae without phagocytes (E.E.R. and B.P.K, unpublished), illustrating the potential for noninvasive visualization of fungal–phagocyte interactions in this highly specialized space in vivo.

### 5.6. Egg

Infection of the zebrafish egg has been investigated as an approach for testing pathogen characteristics [45,133,134,135,136]. While infection at this embryonic stage occurs before the development of many immune system components [119], the potential of this model lies in its applicability to high-throughput screens and studies. Two groups have developed robotic methods for injecting into the yolk of 1–2 hpf zebrafish embryos still within the chorion; one utilized computer image recognition to guide the needle [135] while the other used an agarose grid of dimples to hold the eggs, which naturally aligned themselves with the cellular material to the side, making yolk injection without visual guidance possible [133]. Using a bath infection method at 1 dpf, a recent study found a novel gene, ORF 19.1725, to be involved in *C. albicans* adhesion, penetration, and virulence [137].

### 5.7. Peritoneal Cavity

While this review primarily focuses on the utility of larval zebrafish, adult zebrafish also have advantages as a model. For example, the adaptive immune response to fungal infection can be studied in adult zebrafish [138,139]. In fact, the earliest use of zebrafish for the study of *C. albicans* infection employed intraperitoneal injection of adult fish, which showed that many aspects of *Candida* infection in mice could be replicated in the zebrafish model [44]. These infections have yielded valuable transcriptome data from both host and the pathogen during infection [140]. Combined with existing protein–protein interaction networks, these data have generated hypotheses about host and pathogen interactions at different stages of infection [141,142,143]. Intraperitoneal injections of 4-5 dpf larvae are also easily performed (J.M.D., unpublished).

### 5.8. Muscle and Spinal Tissues

Though they can be more technically difficult, injections into spinal cord tissues can be useful as well. Tenor et al. observed that *Cryptococcus* inoculated into the trunk vacuole (the lumen of the neural tube) replicated unhindered, as neither macrophages nor neutrophils were recruited into this presumably privileged site [58]. This site has also been used to study the mechanisms of demyelination during *Mycobacterium leprae* infection [144]. Injections into the nearby axial muscle can also be used as a model for certain soft tissue infections, although it has been mostly used in adult fish [145,146,147].

## 6. Unique Aspects of the Zebrafish Toolbox: Emerging Techniques and Approaches

While the zebrafish has already fulfilled much of its potential as a unique model host, there remain new ways to take advantage of this unique animal, some of which are only beginning to show their worth. Here we underscore some of the most promising but still relatively untapped approaches to using the model.

### 6.1. High-Throughput Screening for Antifungal Compounds

Antifungal drug development is an urgent need, made difficult by the close evolutionary relationship between fungi and humans. The larval zebrafish is an ideal model in which to assess both in vivo antifungal activity and host toxicity in a complex vertebrate with conserved organ systems and metabolic pathways [148], especially given the fecundity of zebrafish. Additionally, published small molecule screens in zebrafish are able to address organismal-level obstacles faced by many drugs including the pharmacological properties of absorption, distribution, metabolism, and excretion (ADME) [148,149]. Future combinations of small molecule libraries with simple immersion dosing, manual microinjection into easily accessible sites (which can occur at a rate of several hundred per hour), or automated injection systems [134] are a promising direction for antifungal screening. This aspect of the model has already been used to study toxicity in potential antifungals [150].

### 6.2. Imaging Platforms beyond Confocal Microscopy

As detailed in this review, live in vivo imaging in the larval zebrafish has provided valuable insights into localized and whole-body host–fungal interactions. These studies have primarily relied on tried-and-true imaging platforms including epifluorescence, DIC, and confocal microscopy. In the future, state-of-the-art imaging modalities should shed even more light on these interactions.

#### 6.2.1. Light Sheet Fluorescence Microscopy (LSFM)

Light sheet fluorescence microscopy (LSFM) is a burgeoning technology especially well-suited to imaging fungal infections in larval zebrafish. By virtue of its single sheet of illumination, LSFM causes less phototoxicity as only tissues in the focal plane are illuminated, reducing photobleaching and stress to both host and pathogen over extended imaging times. Extended imaging is also made easier through the immobilization of larvae in a lower percentage of agar than what is typically used, creating a less restrictive environment for larval growth and development. Together, these features allow for continued imaging on the order of several days. Thus, using *A. fumigatus* as an example, it could be possible to seamlessly capture conidial swelling, germination, and hyphal expansion in conjunction with innate phagocyte recruitment and dynamics throughout disease progression. While an in-depth explanation of LSFM falls beyond the scope of this review, there are great resources covering LSFM setup, function, and application [151,152,153].

#### 6.2.2. Correlative Light and Electron Microscopy (CLEM)

Combining the live, serial imaging possible with light microscopy with the exquisite ultrastructural detail produced by electron microscopy allows identification of specific host cells and microbes while characterizing their subcellular structures and actions. Techniques for this have been developing in the zebrafish and are beginning to shed light on complex biological processes such as hematopoiesis [154] and angiogenesis [155]. This approach has more recently been applied to studies of infectious pathogenesis in mycobacterial infection models, better defining the interactions of infected macrophages and endothelial cells and the role of autophagy in bacterial clearance [156,157]. Similar studies of fungal infections in larval zebrafish could illuminate previously unknown cellular processes.

#### 6.2.3. High-Throughput Image Analysis

Again because of their large clutch sizes and ease of injection, larval zebrafish are an ideal model in which to perform serial microscopy on large numbers of infected larvae in a multi-well plate or similar device. Even concomitant imaging analyses of 5 to 10 larvae are a remarkable improvement over the options available for in vivo imaging of mammalian model organisms. As demonstrated in one study of cryptococcal infection [56], larvae can be anesthetized and aligned in 96 well plates for repeated imaging up to 24 hpi. Other methods have been used to immobilize larvae for days of imaging, usually by embedding in low melting point agarose and carving out space to allow for linear growth [158]. More recently, dedicated mounting slides have been published which allow rapid, stable mounting of many larvae for specific imaging tasks [159,160]. These systems help to stabilize larvae in specific positions but are not well suited for long term imaging over 24 hours. Other commercial systems can automate repeated larval handling and imaging for a variety of applications [161]. Infectious burden can also be estimated simply through quantification of fluorescent signal produced by a pathogen, a technique that has been successfully applied to infections with *C. albicans* [105], the bacteria *Mycobacterium marinum* [133,162] and in large scale drug efficacy studies [29,53,163,164].

### 6.3. Zebrafish Infection Beyond the Larval Stage

While adult zebrafish generally lack the transparency and ruggedness required for extensive in vivo imaging, adults still have the potential to expand on the biology discovered using larvae. For example, recent work with mycobacteria in adult zebrafish cements and amplifies findings in larvae about granuloma ultrastructure and gene expression [165]. For fungi, adult infections have been useful in examining the genome-wide expression patterns of both *C. albicans* and the host during intraperitoneal infection [44,140,166]. Adult experiments offer an excellent way to assess the contribution of adaptive immunity and confirm the validity of experiments in larvae, as has been done for *C. albicans* infections [138]. Infection of larvae which are then observed beyond the larval period should also be possible but is technically challenging. While these experiments past 10 dpf would require intensive feeding schedules and special considerations in terms of animal care and use, studies on the impact of high fat diets on inflammation and macrophage biology have already begun to solve these problems [167,168]. Needless to say, experimental infections in this period could also shed light on the roles of nutrition and immunological development in host–pathogen interactions.

### 6.4. Analysis and Mathematical Modeling of Large Infection Datasets

The combination of using large numbers of individuals and the ability to gather daily measures of infection in vivo allows for the construction and use of large datasets of many infections in the same experiment over time. Two examples of this have been referred to already here but deserve further emphasis. First is the example of Bojarczuk et al. in the analysis of *Cryptococcus*–phagocyte interactions [56]. Using high-throughput imaging of many infected individuals, the authors were able to analyze major trends in phagocytosis, cryptococcal growth, and macrophage survival. Taking the approach a step further, Ingelsfield et al. applied an array of mathematical modeling techniques to probe the relative importance of different factors in *M. circinelloides* infection [60], such as the rates of phagocyte recruitment, maximum number of phagocytes recruited over time, rate of fungal germination and the strength of germination inhibition by host cells. This study represents an excellent example of what the zebrafish model can contribute to our conceptual understanding of host–pathogen interactions—hopefully there will be more like it to come.

## 7. Conclusions

The zebrafish model has proven extremely valuable in the study of a wide variety of infection types, including the area of fungal pathogenesis. In this review, we have attempted to provide an overview of how this model has been applied to major fungal pathogens, where its unique contributions have been, and what new ways for exploiting this model appear most promising. The zebrafish offers an economical alternative to mammalian studies, excellent visual and molecular tools, and an intriguing gateway to the possibilities of in silico modeling of host–pathogen interactions. Like any animal model it has certain limitations, and is best used with an awareness of those limitations and of what questions the model is capable of answering. Hopefully this review has provided a valuable tour of the limitations and advantages, and will help equip new researchers to use zebrafish in their own work or to interpret and absorb the findings of others.

## Figures and Tables

**Table 1:** Forms of zebrafish larval immunosuppression relevant to fungal infections.

Immunosuppression	Target Gene/Pathway
Morpholino	pu.1 (also referred to as spi1). Can prevent macrophages only or all phagocytes depending upon dose [21]
Morpholino/Mutant	irf8 [23]
Morpholino	csf3r (g-csfr) – neutrophil “depletion” [27]
Morpholino	p47-phox [28]
Morpholino	Duox [28]
Transgenic	Rac2D57N [25,29]
Transgenic	CXCR4b-WHIM [26]
Drug	diphenyleneiodonium (DPI: pan=NADPH Oxidase inhibitor [28]
Drug	FK506 (Calcineurin inhibitor) [30]
Drug	PI3K inhibitor [29]
Drug	CXCR2 inhibitor [29]
Drug	Dexamethasone [31,32]
Clodronate liposomes	Macrophage depletion [22,33]
Transgenic/drug	Metronidazole-induced gene expression [29,32]
Host mutation	Rac2^−/−^ [31]
Host mutation	Mpx^−/−^ [34]

**Table 2:** Fungal mutants in experimental zebrafish and murine infections.

Species	Gene	Larval Zebrafish Reference	Murine Reference
*A. fumigatus*	*sidA*	[27]	[66]
	*laeA*	[27]	[67]
	*aceA*	[68]	[68]
	*metR/Z* overexpression	[69]	[70]
*C. neoformans*	*cap59*	[56]	[71]
	*cap64*	[58]	[72]
	*plb1*	[58]	[73]
	*tps1*	[58]	[74]
	*ure1*	[58]	[75]
	*fnx1*	[58]	[76]
*C. albicans*	*efg1/cph1*	[28,44]	[77]
	*hgc1*	[44]	[78]
	*eed1*	[28,42]	[79,80]
	*NRG1* overexpression	[48]	[81]
	*UME6* overexpression	[29,48]	[81]
	*CAY4975* (white)	[82]	[83]
	*CAY4986* (opaque)	[82]	[83]
	*Oxyellow*	[28]	[84]

**Table 3:** Fungal strains used in zebrafish.

Species	Strain/Isolate
*A. fumigatus*	Af293 [27,31,103]
	CEA10 [31,35]
	ISSFT-F21 [35]
	IF1SW-F4 [35]
	ATCC46645 [36,38]
*C. neoformans*	H99 [56,57,104]
*C. deneoformans*	B3501/2 [57]
*C. albicans*	SC5314 [42,44]
	ATCC 10231 [44]
	*ARG3-GFP*; *ENO1-dTomato* [55]
	*Hwp1-gfp*; *ENO1-dTomato* [105,106]
*M. circinelloides*	CBS277.49 [32,61]
	NRRL3631 [32,61]
*T. marneffei*	*acuD:RFP* (derivative of FRR2161) [12]
